# Supplementary material for: Computational Analysis of Insulin-Glucagon Signalling Network: Implications of Bistability to Metabolic Homeostasis and Disease states
Source: Sci Rep. 2019 Oct 25;9:15298. doi: 10.1038/s41598-019-50889-4 (PMC6814820; doi:10.1038/s41598-019-50889-4)
Supplement: Supplementary file 1 — Supplementary Information [file 41598_2019_50889_MOESM1_ESM.pdf]

SUPPLEMENTARY INFORMATION

Computational Analysis of Insulin-Glucagon Signalling Network: Implications of Bistability to Metabolic Homeostasis and Disease states

Pramod R Somvanshi[a,b], ManuTomar[a] and Venkatesh Kareenhalli[a #]

[a]Department of Chemical Engineering, Indian Institute of Technology, Bombay, Powai, Mumbai, India
[b]Bioengineering Division, John A. Paulson School of Engineering and Applied Science, Harvard University, Cambridge, USA

[#]Corresponding Author: Prof. K V Venkatesh
Department of Chemical Engineering, Indian Institute of Technology, Bombay, Powai, Mumbai, India.

Email - venks@iitb.ac.in
Tel: 91 (22) 2576 7223,
Fax: 91 (22) 2572 6895

Description of Content

Appendix I- Insulin and glucagon signalling pathway

Appendix II- Details on mathematical modelling (parameters and equations)

Appendix III- Supplementary Figures

Appendix IV- Sensitivity analysis

Appendix-I : Description of Insulin and Glucagon Signaling Pathways

A. Insulin signaling

Insulin signalling is brought about by a complex network of synchronised events. Insulin binds to its cell surface receptor (IR) and induces a conformational change on the receptor leading to its activation[1,2]. On activation, the insulin receptor (IR) phosphorylates several members of the insulin receptor substrate (IRS)[3]. IRS triggers activation of PI3K and conversion by its catalytic domain of phosphatidylinositol (3,4)-bisphosphate (PIP_2) lipids to phosphatidylinositol (3,4,5)-trisphosphate (PIP_3). PKB/AKT binds to PIP_3 at the plasma membrane and subsequently gets activated. This PI3K-AKT pathway is highly conserved in most of the metabolic actions of insulin and is a critical node in the insulin signalling pathway[4,5]. PI3K also activates PKC[6] which negatively regulates the function of IRS proteins[7–9]. Similar findings suggest AKT to be a positive regulator of IRS activation[10,11]. Further, AKT and PKC activate GLUT4 translocation to the cell surface[12,13] subsequent to which glucose is transported inside the cell. Similarly, stimulated by insulin, fatty acid transport protein (FATP) and amino acid transport proteins (AATP) play a role in absorption of fatty acids and amino acids, respectively[14,15] and initiate the anabolic processes in cells when the concentration of these metabolites become excess in plasma. Moreover, activation of mTOR pathways is essential for protein synthesis which is regulated by insulin & amino acids[16]. Insulin regulates the activation of mTOR through the action of AKT whereas amino acids directly activate mTORc2 complex that phosphorylates S6K1 and further activates it for protein synthesis. S6K1p exerts a negative feedback on insulin signalling by serine phosphorylation of IRS1 adaptor molecule and also inhibition of formation of mTOR-rictor complex which in turn activates AKT[17,18]. Moreover, AKT also activates PDE3 which enhances degradation of cAMP mediated glucagon signalling[19].

B. Glucagon signalling

Glucagon, on the other hand, acts opposite to insulin action both at the signalling and secretion levels. It initiates the breakdown of glycogen and triglyceride stores for glucose release from muscle & liver and fatty acid release from adipose tissues, respectively, during exercise and fasting conditions[20]. Glucagon signalling mechanisms trigger metabolic pathways via secondary messengers like cyclic adenosine monophosphate (cAMP), cAMP dependent protein kinase (PKA) & calcium. Glucagon binds to the G protein coupled receptors (GPCR) present on the cell surface; thereby altering the conformation of the cytoplasmic domain of GPCR. Activated GPCR binds to trimeric GTP binding proteins (G proteins) via GDP-GTP exchange which relays the intracellular signal downstream to G-protein linked receptors[21–24]. Gs and Gq are the two different kinds of G proteins associated with the glucagon receptors which relay two distinct signalling pathways. Gs protein activate the adenylate cyclase enzyme which leads to the increase in cAMP concentration that binds and activates PKA in the cells[25]. Gq proteins activate PLC that activates the secondary messenger IP3, which in turn stimulates the release of intracellular calcium from the endoplasmic reticulum stores. Both activation of glycogen phosphorylase and increase in calcium levels promote glycogen breakdown in muscle and liver. PLC hydrolyses the membrane phospholipid PIP_2 to form IP_3 and diacylglycerol (DAG). DAG recruits PKC attaching it to the plasma membrane, whereas IP_3 diffuses to the ER and binds to an IP_3 receptor that serves as a Ca^{2+} channel and releases calcium ions from the ER into the cytosol[26]. PKC interacts with DAG via its C1 domain by undergoing a conformational change, thus enabling it to phosphorylate one of its substrate IRS and thereby inhibiting insulin signalling and subsequent glucose uptake[27].

Appendix II : Details of the Mathematical Model

Table 1. List of parameters

Parameter	Value	Units	Parameter	Value	Units
n_{akt}	1.8	-	$Glcn\_{recp}$	9*1E-13	M
n_{pkc}	1	-	a_4	6*1E7	/(M.min)
k_{akt}	4.25	%	a_3	0.1	/min
k_n	7	%	R_t	126500	-
$k1a_{Basic}$	137775	/min	a_{lf}	10000	-
$k1_f$	673.445*E10	/min	V	11	L
$k1_b$	543752	/min	V_p	3	L
$k2_f$	141.254	/min	c_1	10*60	/min
$k21_f$	9178.23*7	/min	c_2	100*60	/min
$k2_b$	3533330	/min	c_{3a}	312*1E -3	/min
$k4_f$	226300	/min	c_4	4*60*1E-3	/min
$k4_b$	1493960	/min	c_5	5.2*60*1E-3	/min
$k4_{f1}$	513844	/min	c_8	650*60	/min
$k4_{b1}$	320822	/min	A_0	3	AU
$k6_f$	1337290/2	/min	B_1	100	AU
$k6_b$	1629.22*20	/min	B_2	1E6	AU
$k5_f$	0.06308	/min	k_{fdag}	2*60*0.01	/min
$k5_b$	0.212	/min	v_f	60	/min
$k8_f$	856353	/min	k_{bdag}	0.5*60	/min
$k8_{f1}$	856353/2	/min	k_{c1}	2.0*1E -6	μM /min
$k8_b$	1430390*1.5	/min	k_{cm1}	25*1E -12	M
$AMPK\_{eff}$	AMPK	AU	k_{c2}	1.18	/min
$PP2A_{max}$	5	M	k_{cm2}	1	AU
f_{20}	1.5E-3	/min	p_n	1	-
f_{21}	5E-4	/min	p_e	2	-
n_s	3	-	c_{qi}	2	AU
f_{aa}	0.75	mM	V_{a1}	0.9	/min
V_i	0.05	l/kg	V_{a2}	8	/min
m_1	0.19	/min	k_{camp1}	2*3.2*1E -6	μM
m_2	0.484	/min	k_{camp2}	4*3.2*1E -6	μM
m_4	0.194	/min	PKA_t	6*1E-4	μM
m_5	0.0304*Vi	/min	K_{akt1}	0.3	/min
m_6	0.6471	-	k_{pde}	1.5	/min
$Gamma$	0.5	/min	$PDE3_t$	5	AU
$AMPK_t$	1	AU	PKA_L	PKA/(8*1E-6)	-
k_{am1}	1	mM	k_s	1E-4	μM
K_{am2}	2.25	mM	K_{bpde3}	0.001	/min
a_1	0.12*1.25	/min	K_{AMP_L}	0.16	mM
a_2	0.3	/min	K_{ATP_L}	2.8	mM
G_m	1.35*1E-10	M/min	K_{mpr}	2	AU
p_1	0.005*180	/(mg/L)	Bs_{Ala}	0.25	mM
q_1	10	-	V_{ala}	2.50E-10	M
V_{glu}	48*E-12	M/min	V_{gprt}	26	AU/min
n_g	4.65	-	k_{lrs}	95000	AU
km_g	8.9	mM	V_{plc}	233	AU/min

km_a	0.6	mM	k_{gprt}	32	AU
k_{mf}	1.6	mM	V_{ip3}	16.3	AU/min
n_a	5.8	-	k_{plc}	42	AU
n_f	4.8	-	V_{cal}	35	AU/min
V_{aa}	25*E-12	M/min	k_{ip3}	9	AU
V_{fa}	25*E-12	M/min	K_{gcn}	3.20E-05	M
V_{sk}	0.2	AU	K_{mak}	0.1	%
K_{msk}	6	AU	K_{pka}	12	AU
K_{mfa}	1.5	mM	V_{akn}	5	AU
K_{mdg}	7	mM	K_{man}	0.05	%
V_{mt}	2.5	AU	K_{ipk}	3	AU
V_{pi3}	3	AU	K_{mp2}	6	%
K_{mp3}	4	%	K_{mgln}	25E-12	M
V_{gcn}	2	AU			

Table 2. List of State Variables

Sr. No.	State Variables	Full Form
1	IR	Insulin Receptor
2	IRp	Insulin Receptor Phosphate
3	IRS	Insulin Receptor Substrate
4	IRSp	Phosphorylated Insulin Receptor Phosphate
5	PI3K	Phosphoinositide 3-kinase
6	PI3Kp	Phosphorylated Phosphoinositide 3-kinase
7	AKT	Protein Kinase B
8	AKTp	Phosphorylated Protein Kinase B
9	PKC	Protein Kinase C
10	PKCp	Phosphorylated Protein Kinase C
11	GLUT4c	Glucose Transporter type 4 (cytosol)
12	GLUT4s	Glucose Transporter type 4 (surface)
13	mTOR	Mammalian target of rapamycin
14	mTORa	Activated mammalian target of rapamycin
15	S6K1	Ribosomal protein S6 kinase beta-1
16	S6K1a	Activated Ribosomal protein S6 kinase beta-1
17	InsV	Insulin concentration in portal vein
18	InsL	Insulin concentration in Liver
19	InsP	Insulin concentration in Plasma
20	GlnP	Glucagon in plasma
21	Glcn	Glucagon in cell
22	FR	Free Recptor
23	RS	Sequestored Receptor
24	LR	Ligand Bound Receptor
25	LRS	Ligand Bound Sequestered Receptor (GPCR)
26	LRP	Desensitized Ligand Receptor Complex

27	GPRT	G Protein
28	PLC	Phospholipase C
29	IP3	Inositol tri Phosphate
30	DAG	Diacyl glycerol
31	PKA	Protein Kinase A
32	cAMP	Cyclic Adenosine Monophosphate
33	PDE3a	Phosphodiesterase 3
34	AMPK	AMP activated Protein Kinase
35	Cal	Calcium

A. Model Equations with comments

Insulin secretion kinetics

Insulin secretion in response to the levels of plasma glucose, alanine and free fatty acids in plasma.

$$Insec = Vglu * \left(\frac{Ca_{glu}^{ng}}{Ca_{glu}^{ng}+kmg^{ng}}\right) + Vaa * \left(\frac{Ca_{ala}^{na}}{Ca_{ala}^{na}+kma^{na}}\right) + Vfa * \left(\frac{Ca_{ffa}^{nf}}{Ca_{ffa}^{nf}+kmf^{nf}}\right)$$

(Scaled up insulin secretion rate by pancreas)

$$ISR = Insec/10^{-12}$$

Insulin in portal vein

$$\frac{d(Ins_V)}{dt} = -(gamma * InsV) + ISR$$

$$InSec = gamma * InsV$$

HE-Hepatic Extraction of Insulin

Total insulin flux leaving the liver[28]

$$HE = (-m5 * InSec) + m6$$

$$m3 = (HE * m1)/(1 - HE)$$

Insulin in Liver

$$\frac{dIns_L}{dt} = -(m1 + m3) * InsL + m2 * InsP + InSec$$

Insulin in Plasma

$$\frac{dIns_P}{dt} = -(m2 + m4) * InsP \; + \; m1 * InsL$$

Effect of glucagon on plasma insulin

Glucagon has inhibitory effect on plasma insulin levels

$$insulin_p = \frac{Ins_p * 10^{-12}}{Vi} * Vgln * \frac{Kmgln^2}{Gln_p^2 + kmgln^2}$$

Insulin signalling kinetics

Rate of phosphorylation of IR with insulin as well as without insulin

$$v_{1f} = k_{1f} * insulin_p * 1000 * IR + K_{1aBasic} * IR$$

Rate of dephosphorylation of IR_p

$$v_{1b} = k_{1b} * IR_p$$

Rate of phosphorylation of IRS_p which is activated by AKT_p and inhibited by PKC_p

$$v_{2f} = k_{2f} + k_{21f} * \left(\frac{AKT_p^{n_{akt}}}{AKT_p^{n_{akt}} + k_{akt}^{n_{akt}}}\right) * IRp * IRS * \frac{k_n}{k_n + \left(\frac{PKC_p}{1.5}\right)^{n_{pkc}}}$$

S6K negative feedback on IRS1

$$S6K_{Ntv} = Vsk * (\frac{S6K1a^4}{kmsk^4 + S6K1a^4})$$

Rate of dephosphorelation of IRS_p

$$v_{2b} = k_{2b} * IRS_p * (1 + S6K_{Ntv})$$

Rate of dephosphorylation of PI3K by IRS_p

$$v_{4f} = k_{4f} * PI3K * IRS_p$$

Rate of phosphorylation of PI3K

$$v_{4b} = k_{4b} * PI3K_p$$

Rate of phosphorylation of AKT by $PI3K_p$

$$v_{4f1} = k_{4f1} * AKT * PI3K_p$$

Rate of dephosphorylation of AKT_p

$$v_{4b1} = k_{4b1} * AKT_p$$

Positive feedback of FFA on PKC

$$FFA_{ptv_{pkc}} = 1 + 0.5 * \left(\frac{FFA^3}{FFA^3 + kmfa^3}\right)$$

Positive feedback of DAG on PKC

$$DAG_{ptv_{pkc}} = 1 + \frac{DAG^3}{DAG^3 + kmdg^3}$$

Rate of phosphorylation of PKC by $PI3K_p$, it is activated positive feedbacks from free fatty acids (FFA) and DAG

$$v_{6f} = k_{6f} * FFA_{ptv_{pkc}} * DAG_{ptv_{pkc}} * PKC * PI3K_p$$

Rate of dephosphorylation of PKC_p

$$v_{6b} = k_{6b} * PKC_p$$

Rate of translocation of GLUT4 from cytosol to cell surface, activated by AKT_p and PKC_p

$$v_{5f} = k_{5f} * \left(0.2 * AKT_p + 0.8 * PKC_p\right) * GLUT4_c$$

Rate of translocation of GLUT4 from cell surface to cytosol

$$v_{5b} = k_{5b} * GLUT4_s$$

mTOR Raptor activation by AA in absence of insulin

$$AA_{mTOR_{eff}} = Vmt * \frac{AA^{ns}}{AA^{ns} + faa^{ns}}$$

Rate of mTOR activation by AKT_p and amino acids (AA)

$$v_{8f} = k_{8f} * mTOR * AKT_p + k_{8f1} * \left(1 + AA_{mTOR_{eff}}\right) * mTOR$$

Rate of $mTOR_a$ deactivation

$$v_{8f} = k_{8b} * mTORa$$

S6K1 phosphorylation-dephosphorylation

$$PDK1_a = Vpi3 * \frac{PI3K}{Kmp3 + PI3K}$$

$$PP2_a = PP2_{amax} * \frac{Kmp2^{1.2}}{Kmp2^{1.2} + mTORa^{1.2}}$$

$$AMPK_{Ntv_{S6K}} = \frac{0.75^2}{0.75^2 + AMPK_{eff}^2}$$

$$reg_1 = \frac{kmpr^4}{kmpr^4 + PP2_a^4}$$

Rate of S6K1 phosphorylation; activated by PI3K & $mTOR_a$, inhibited by $PP2_a$ and AMPK

$$v_{9f} = f_{20} * PDK1_a * mTOR_a * reg_1 * S6K1 * AMPK_{Ntv_{S6k}}$$

Rate of $S6K1_a$ dephosphorylation

$$v_{9b} = f_{21} * PP2_a * S6K1_a$$

ODEs: Mass balance equations

$$\frac{d(IR)}{dt} = v_{1b} - v_{1f}$$

$$\frac{d(IR_p)}{dt} = v_{1f} - v_{1b}$$

$$\frac{d(IRS)}{dt} = v_{2b} - v_{2f}$$

$$\frac{d(IRS_p)}{dt} = v_{2f} - v_{2b}$$

$$\frac{d(PI3K)}{dt} = v_{4b} - v_{4f}$$

$$\frac{d(PI3K_p)}{dt} = v_{4f} - v_{4b}$$

$$\frac{d(AKT)}{dt} = v_{4b1} - v_{4f1}$$

$$\frac{d(AKT_p)}{dt} = v_{4f1} - v_{4b1}$$

$$\frac{d(PKC)}{dt} = v_{6b} - v_{6f}$$

$$\frac{d(PKC_p)}{dt} = v_{6f} - v_{6b}$$

$$\frac{d(GLUT4_c)}{dt} = v_{5b} - v_{5f}$$

$$\frac{d(GLUT4_s)}{dt} = v_{5f} - v_{5b}$$

$$\frac{d(mTOR)}{dt} = v_{8b} - v_{8f}$$

$$\frac{d(mTOR_a)}{dt} = v_{8f} - v_{8b}$$

$$\frac{d(S6K1)}{dt} = v_{9b} - \mathrm{v}_{9f}$$

$$\frac{d(S6K1_a)}{dt} = v_{9f} - v_{9b}$$

Glucagon Secretion and kinetics

$$if\ (Ca_{Ala} - bs_{Ala}) >= 0\ ;\ AmGlcn\ =\ (Ca_{Ala} - bs_{Ala});\ else\ \ AmGlcn = 0$$

Glucagon secretion is inhibited by glucose and activated by amino acids

$AA_{Glcn_{eff}} = Vala * \left(\frac{Am_{Glcn}^{4.5}}{Am_{Glcn}^{4.5}+1}\right)$; *if Ca_Glu <=5;*

$$Glcn_{Sec} = \frac{Gm}{\frac{Ca_{Glu}}{5} + q_1 * \exp\left(p_1 * (Ca_{Glu} - 5)\right)} + AA_{Glcn_{eff}};\ else$$

$$Glcn_{Sec} = \frac{Gm}{1+q_1*\exp\left(p_1*(Ca_{Glu}-5)\right)} + AA_{Glcn_{eff}}$$

Plasma Glucagon conc.

$$\frac{dGln_p}{dt} = -(a1 + a2) * Gln_p + Glcn_{Sec}$$

Glucagon receptor signalling model

Due to the faster timescales of the calcium signalling with respect to other signalling events, the Hill formulations have been used under pseudo steady state approximations in order to describe the input-output relationship in form of dose response curve for the mentioned below 4 signalling components – GPRT, PLC, IP3i, Cal

$$GPRT = Vgprt * \frac{LRS}{klrs + LRS}$$

$$PLC = Vplc * \frac{GPRT^2}{kgprt^2 + GPRT^2}$$

$$IP3i = Vip3 * \frac{plc^4}{kplc^4 + plc^4}$$

$$Cal = Vcal * \frac{IP3i^{1.8}}{kip3^{1.8} + IP3i^{1.8}}$$

$$\frac{dGlcn}{dt} = a_1 * V_p * \frac{Glnp}{V} - a_3 * Glcn - a_4 * Glcn * \frac{FR}{Rt} * Gln_{recp}$$

$$GCN = Glcn * 10^6$$

$$c_3 = c_{3a} * Vgcn * \frac{GCN}{kgcn + GCN}$$

FR-free receptor

$$\frac{dFR}{dt} = c_1 * LR - c_2 * GCN * FR - c_3 * FR + c_4 * RS$$

RS-Sequestered receptor

$$\frac{dRS}{dt} = c_1 * LRS - alf * c_2 * GCN * RS + c_3 * FR - c_4 * RS$$

LR-Ligand bound receptor

$$\frac{dLR}{dt} = c_2 * GCN * FR - c_1 * LR + \frac{c_4}{alf} * LRS - c_3 * LR + c_5 * LRP$$

LRS-ligand bound sequestered receptor (denotes active GPCR)

$$\frac{dLRS}{dt} = c_3 * LR - \frac{c_4}{alf} * LRS + alf * c_2 * GCN * RS - c_1 * LRS - c_8 * \left(1 + A_0 * \frac{GPRT}{B_1 + GPRT} * \frac{LRS}{B_2 + LRS}\right)$$

LRP – desensitized ligand receptor complex

$$\frac{dLRP}{dt} = c_8 * \left(1 + A_0 * \frac{GPRT}{B1 + GPRT} * \frac{LRS}{B_2 + LRS}\right) - c_5 * LRP$$

The above 5 equations describe the dynamic model[29] of glucagon binding to G-Protein Coupled Receptors that includes G protein activation and receptor desensitization.

DAG (Diacylglycerol)

$$Kc2p = 1 - \left(\frac{PKA^4}{ks^4 + PKA^4}\right)$$

$$\frac{dDAG}{dt} = kfdag * Cal * \frac{plc}{K_{c2p} + 0.1 * Cal} + V_f * FA - kbdag * DAG$$

cAMP-PKA signalling (Cyclic Adenosine Monophosphate – Protein Kinase A)

$$Kck = 1 + 0.5 * cqi * \frac{Cal^3}{9^3 + Cal^3}$$

$$Jg12 = Kck * K_{c1} * \left(\frac{Glnp^2}{K_{cm1}^2 + Glnp^2}\right) - kc2 * \frac{PDE3_a}{K_{cm2} + PDE3_a} * cAMP$$

$$Jg13 = V_{a1} * \frac{cAMP^3}{(Kcamp_1)^3 + cAMP^3} * (PKA_t - PKA) - V_{a2} * PKA * \frac{Kcamp_2}{Kcamp_2 + cAMP}$$

PKA (Protein Kinase A)

PKA is activated by cAMP

$$\frac{dPKA}{dt} = Jg13$$

cAMP (cyclic Adenosine Monophosphate)

cAMP is activated by Calcium & Glucagon and is degraded by PDE3 and inhibited by PKA

$$\frac{dcAMP}{dt} = Jg12 - 2 * \frac{dPKA}{dt}$$

PDE3a (Phosphodiesterase 3 activated)

$$AKT_{ptv_{PDE3}} = K_{akt1} * \frac{AKT_p^2}{kmak^2 + AKT_p^2}$$

Rate of activation of PDE3; with positive feedbacks from AKT_p and PKA promoting PDE3 degradation

$$\frac{dPDE3}{dt} = AKT_{ptv_{PDE3}} * (PDE3_t - PDE3_a) - k_{pde} * \frac{PKA_L}{kpka + PKA_L} * PDE3a$$

AMPK (adenosine monophosphate activated protein kinase)

$$AKT_{ntv_{AMPK}} = Vakn * \frac{AKT_p^2}{AKT_p^2 + kman^2}$$

$$PKA_{ntv_{AMPK}} = \frac{kipk^2}{kipk^2 + PKA_L^2}$$

Rate of activation of AMPK; inhibited by AKT_p and PKA

$$\frac{dAMPK}{dt} = k_{am1} * AMP_{ATP_{AMPK}} * PKA_{ntv_{AMPK}} * (AMPK_t - AMPK) - K_{am2} * AMPK * AKT_{ntv_{AMPK}}$$

Appendix III : Figures

A. Model Validation

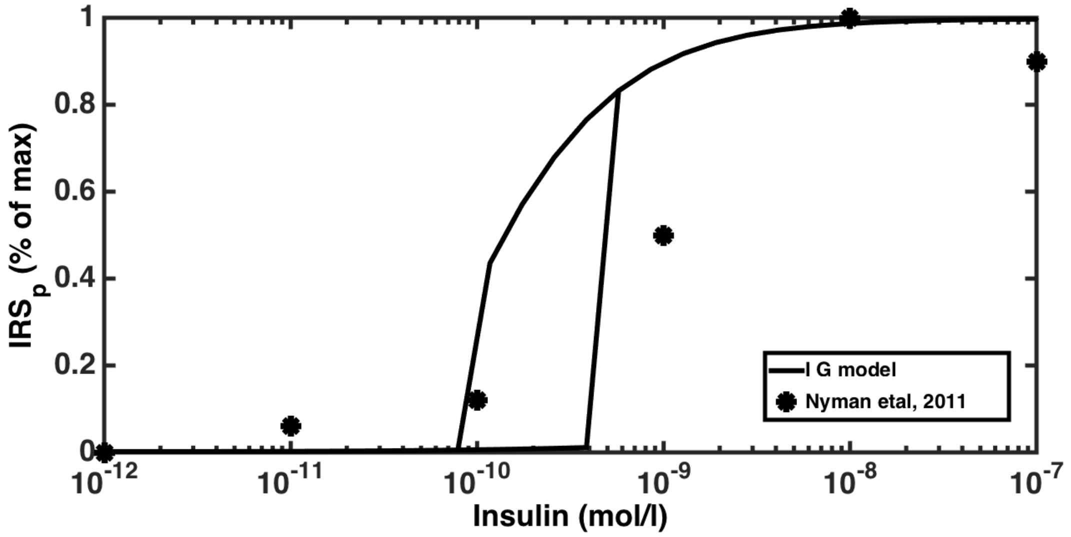

(a)

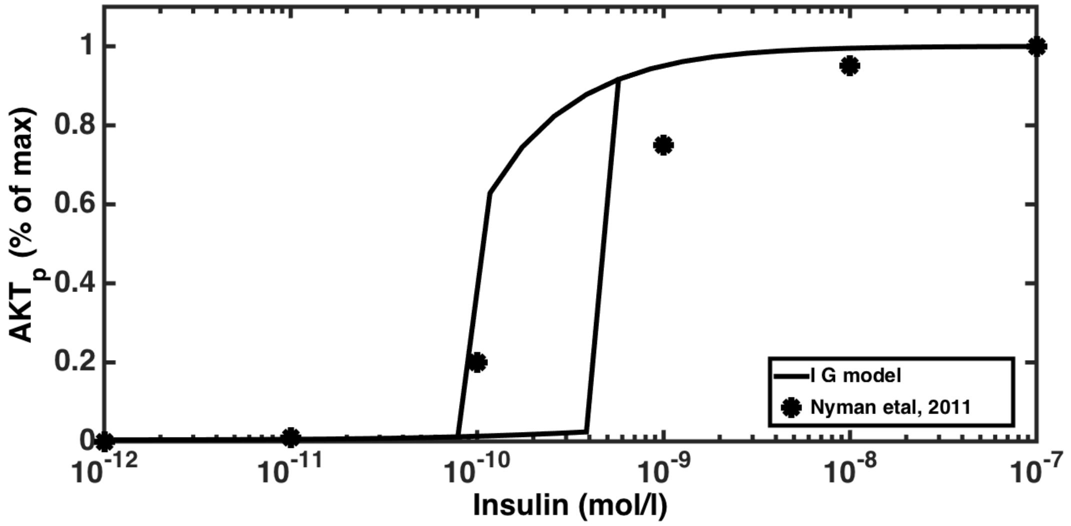

(b)

Figure S1. Model Validation. Dose response to changing concentration of insulin. Solid graph is simulated by our model which shows bistable response for increasing and decreasing levels of insulin. Black filled stars indicate experimental data obtained from the input response to isolated human adipocytes[30] for (a) phosphorelated IRS (b) phosphorelated AKT.

B. Flux Maps

Note : Flux values below 10^{-5} are mentioned as zero in the flux maps (S2-S11) given in this section.

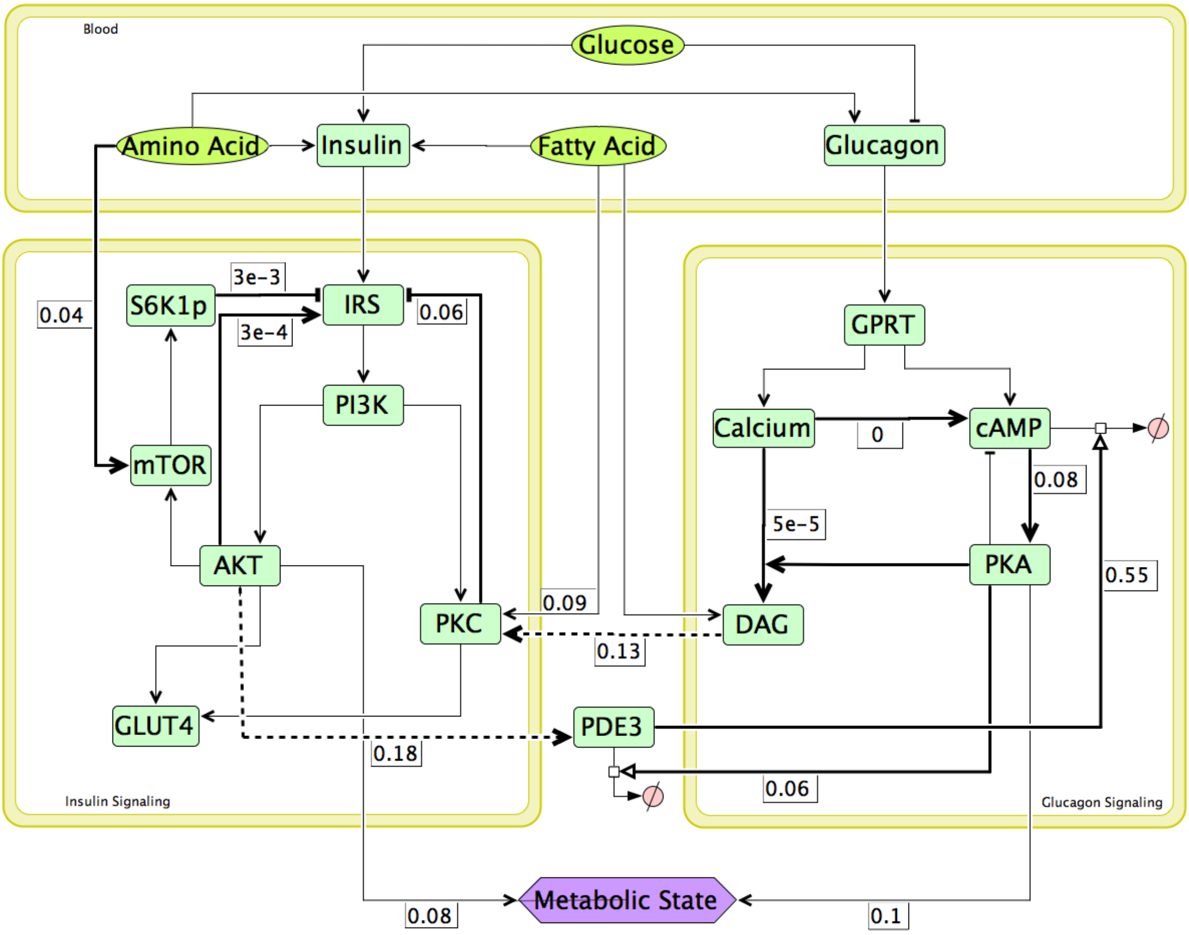

Figure S2. Network map at glucose = 1 fold, AA & FA = 1 fold i.e., during resting conditions. Numbers along the bold lines indicate magnitude of activation or inhibition in terms of the corresponding Hill function values, which lie between 0 and 1. Here most feedbacks & crosstalks are almost inactive except cAMP degradation by PDE3. Overall metabolic state is slightly catabolic with glucagon signalling module being more active than insulin signalling module (AKT contribution = 0.08 and PKA contribution = 0.1).

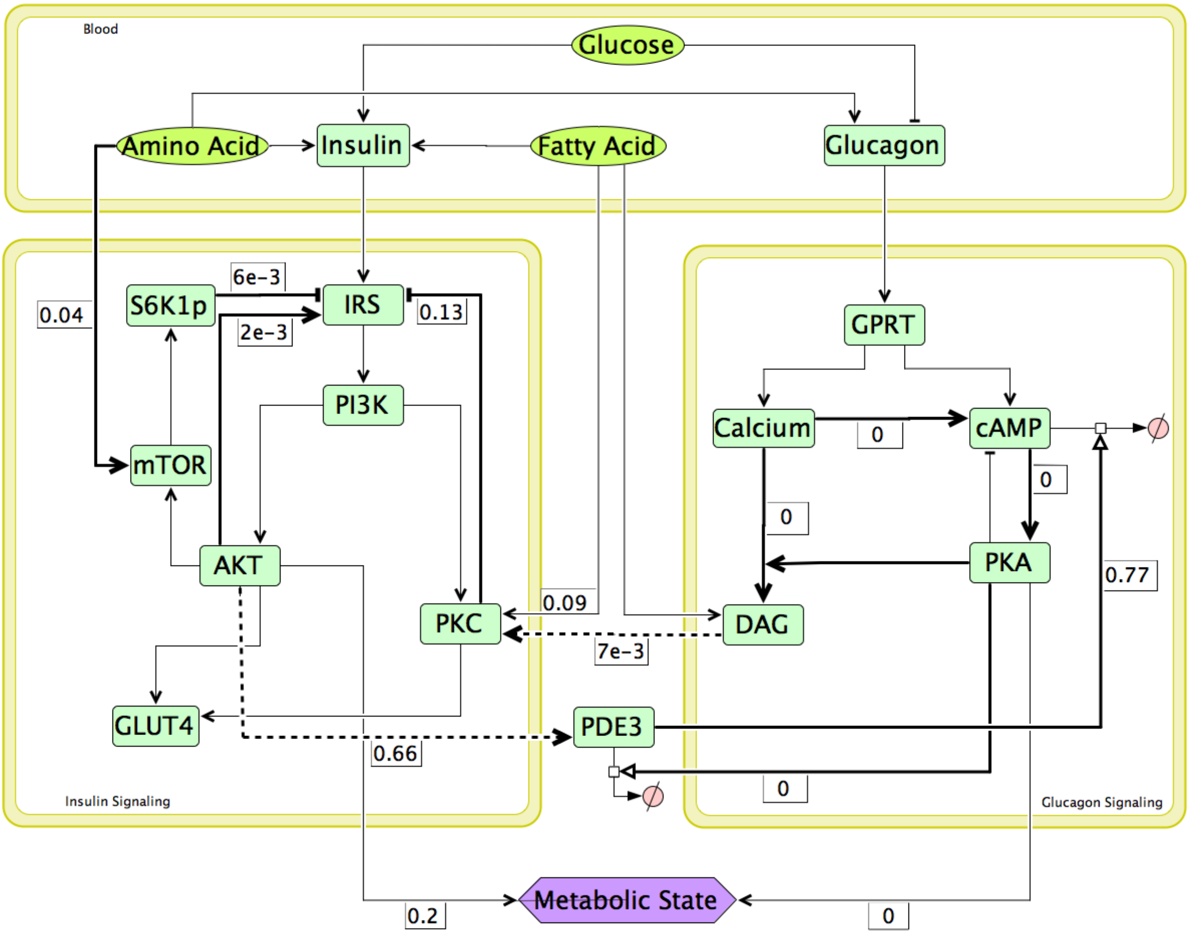

Figure S3. Network map at glucose = 1.5 fold, AA & FA = 1 fold during switching ON conditions. Numbers along the bold lines indicate magnitude of activation or inhibition in terms of the corresponding Hill function values, which lie between 0 and 1. Here AKT activation of IRS has increased slightly (= 2e-3 vs 3e-4 in Figure S2), whereas PDE3 degradation of cAMP has increased (= 0.77 vs 0.55 in Figure S2) as compared to the resting state. Overall metabolic state is mildly anabolic with contribution from insulin signalling being more than glucagon signalling to the net metabolic state (AKT contribution = 0.2 and PKA contribution = 0).

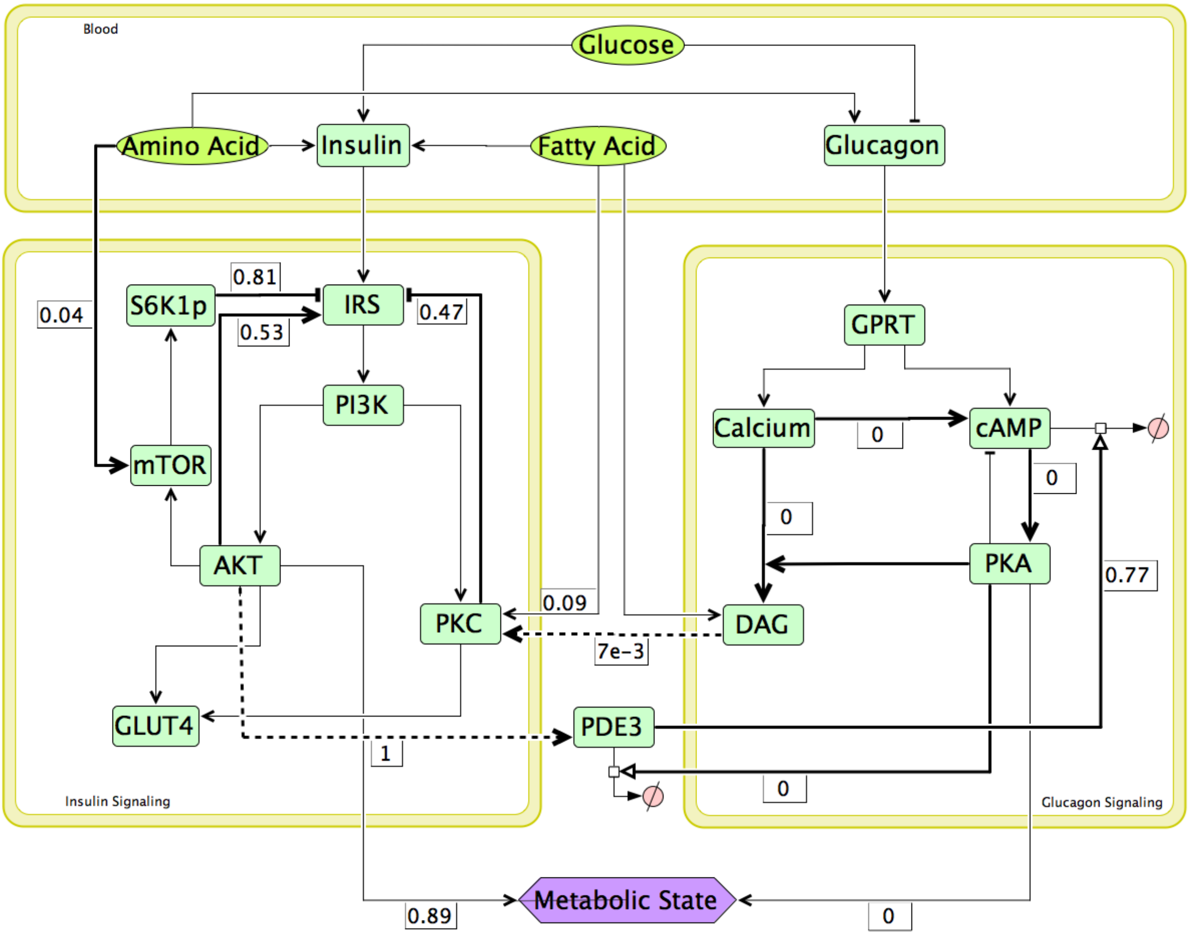

Figure S4. Network map at glucose = 1.5 fold, AA & FA = 1 fold during switching OFF conditions. Numbers along the bold lines indicate magnitude of activation or inhibition in terms of the corresponding Hill function values, which lie between 0 and 1. Here AKT positive feedback on IRS has strengthened (= 0.53 vs 3e-4 in Figure S2) along with the negative feedbacks of S6K1p (0.81 vs 3e-3 in Figure S2), PKC on IRS (0.47 vs 0.06 in Figure S2) and AKT crosstalk PDE3 (= 1 vs 0.18 in Figure S2) compared to the resting state. Overall metabolic state is heavily anabolic with contribution from insulin signalling being much more than glucagon signalling to the net metabolic state (AKT contribution = 0.89 and PKA contribution = 0).

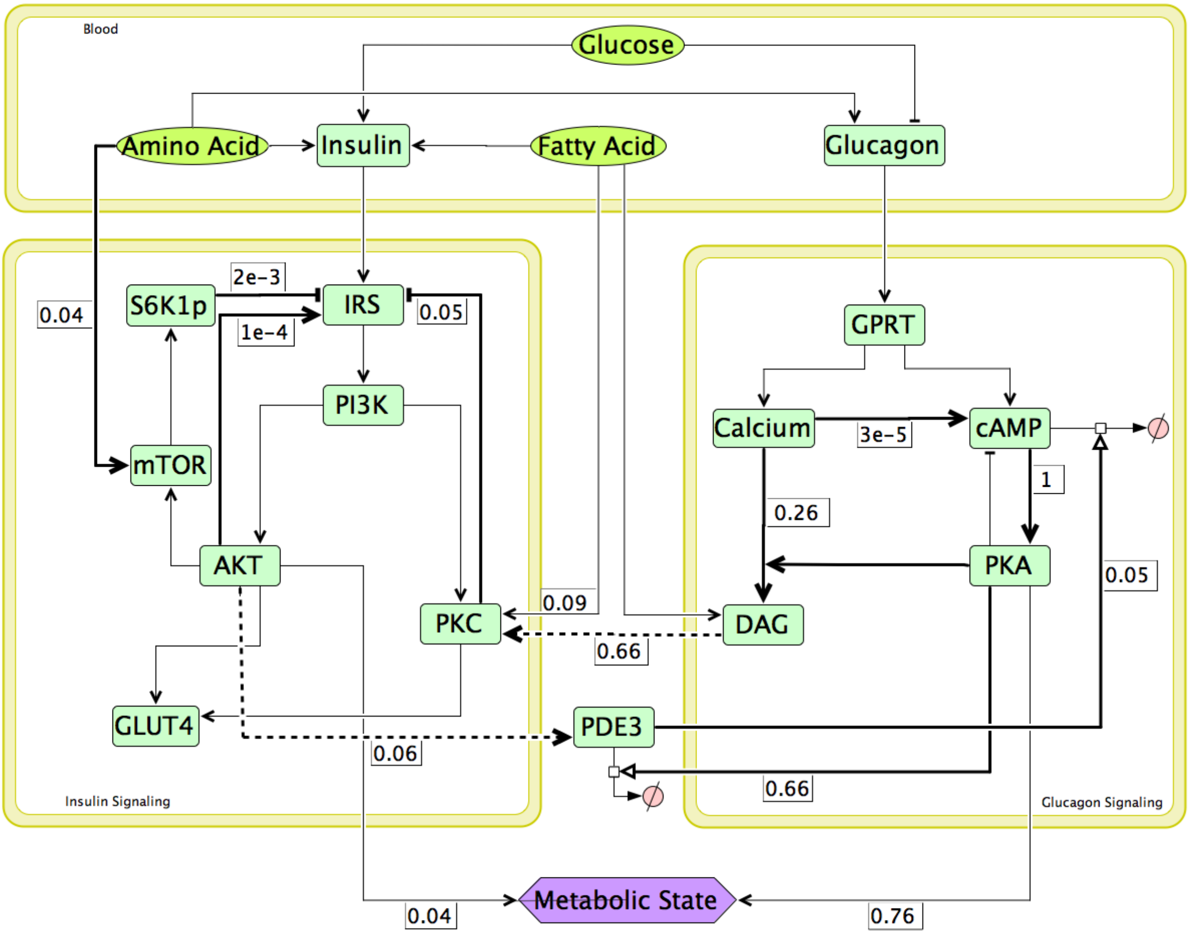

Figure S5. Network map at glucose = 0.8 fold, AA & FA = 1 fold. Numbers along the bold lines indicate magnitude of activation or inhibition in terms of the corresponding Hill function values, which lie between 0 and 1. Here PDE3 degradation of cAMP is minimal (= 0.05 vs 0.55 in figure S2), cAMP activation of PKA is maximum (=1) in this case. Hence, overall metabolic state is heavily catabolic with contribution from insulin signalling being much less than glucagon signalling (AKT contribution = 0.04 and PKA contribution = 0.76).

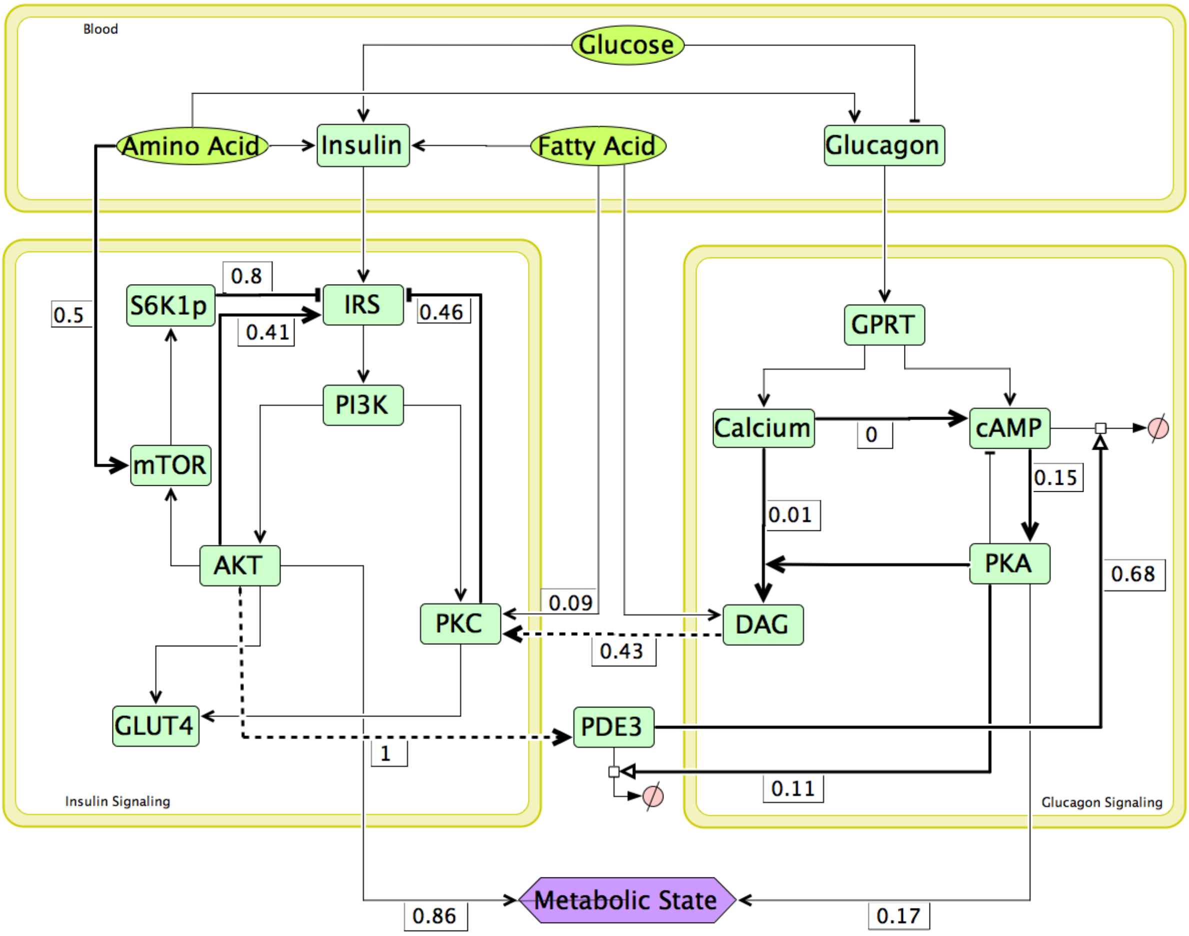

Figure S6. Network map for high plasma AA levels; with AA = 3 folds, glucose = 1 fold & FA = 1 fold for switching OFF conditions. Numbers along the bold lines indicate magnitude of activation or inhibition in terms of the corresponding Hill function values, which lie between 0 and 1. Although high AA levels are inhibiting insulin signalling pathways through increased S6K1p inhibition of IRS (= 0.8), it is also activating IRS-PI3K-AKT pathways (via increasing insulin secretion from pancreas). Moreover, AKTp positive feedback on IRS (= 0.41) and AKT crosstalk PDE3 (= 1) and cAMP degradation (=0.68) are active. Although DAG inhibition on IRS (via PKC) has increased (PKC inhibition on IRS = 0.46 vs 0.06 under resting conditions), it is not able to counter the activating effect of AKT positive feedback on IRS. Hence, overall metabolic state is heavily anabolic with contribution from insulin signalling being much more than glucagon signalling to the net metabolic state (AKT contribution = 0.86 and PKA contribution = 0.05).

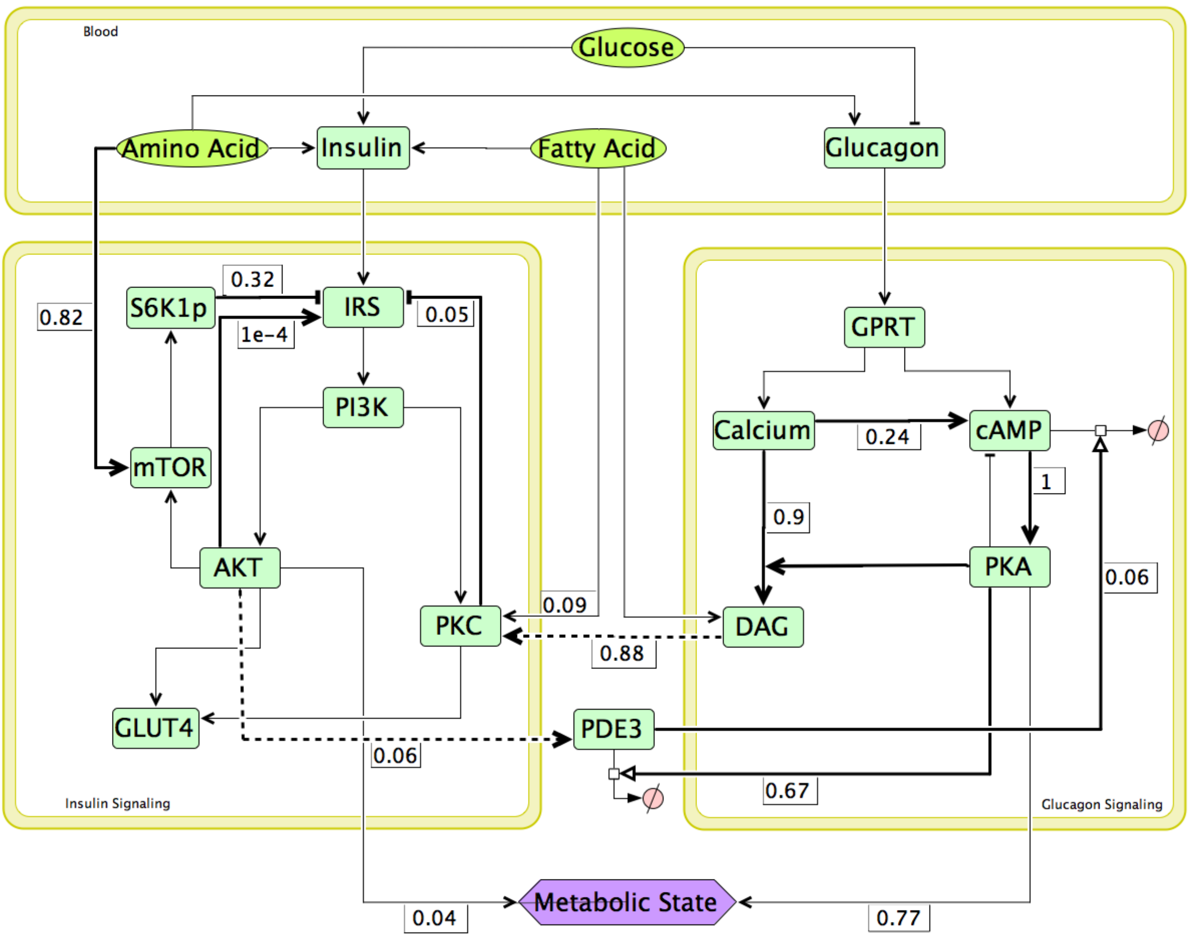

Figure S7. Network map for excess AA and high glucose levels in plasma; with glucose = 1.5 folds, AA = 5 folds & FA = 1 fold. Numbers along the bold lines indicate magnitude of activation or inhibition in terms of the corresponding Hill function values, which lie between 0 and 1. Here activation of mTOR-S6K1p pathway by plasma amino acid is inhibiting IRS as seen from the high strength of AA-mTOR activation (0.82) and S6K1p inhibition IRS (0.32). Whereas Glucagon signalling fluxes are highly activated by high amino acid levels. This leads to Ca-DAG (= 0.9) and cAMP-PKA (= 1) activations becoming very high in spite of inhibitions from high glucose levels in these condition. Insulin signalling module is almost shut off and very low levels of downstream AKTp is unable to degrade cAMP via PDE3 activation (=0.06). Hence, overall metabolic state is heavily catabolic with contribution from insulin signalling being much less than glucagon signalling to the net metabolic state (AKT contribution = 0.04 and PKA contribution = 0.77).

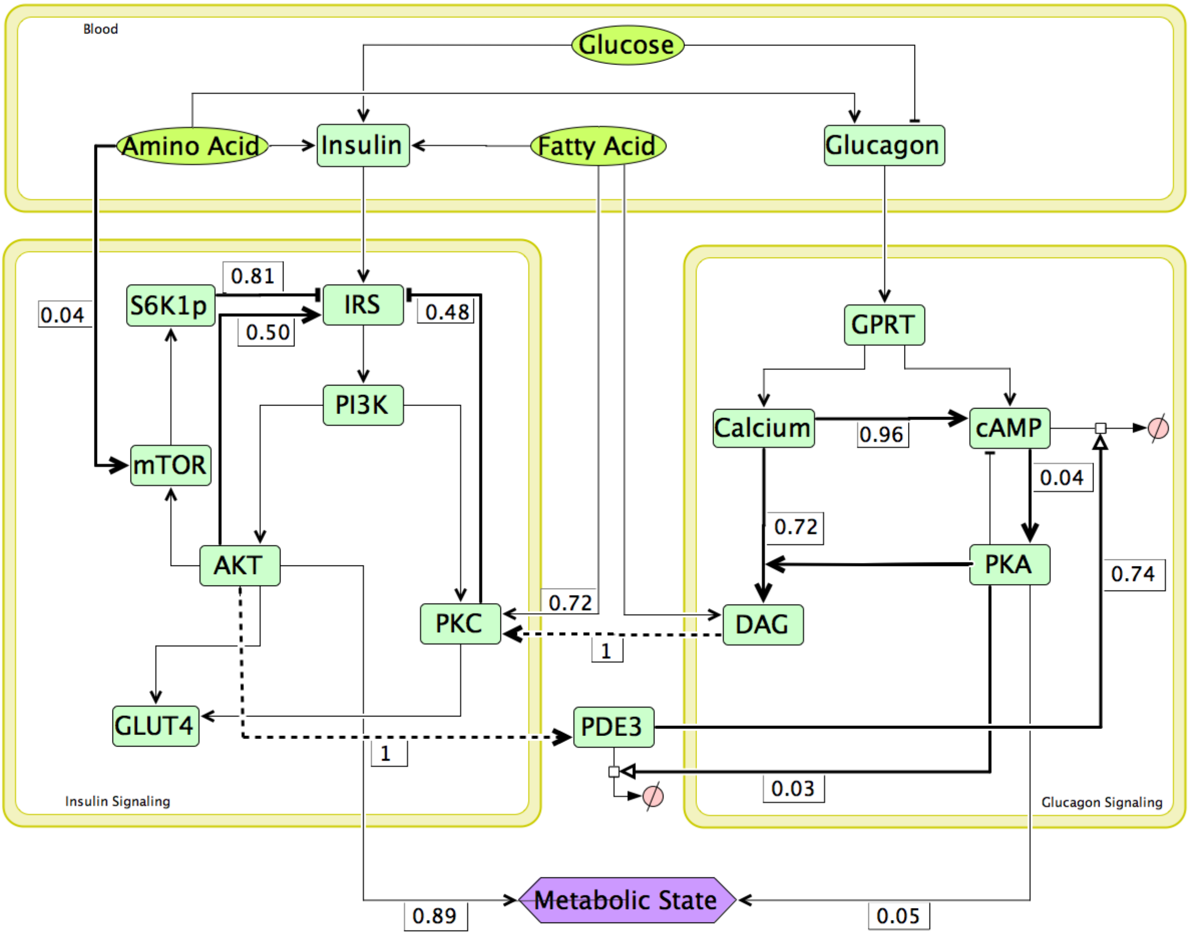

Figure S8. Network map for high FA levels in plasma; with FA = 3 folds, glucose = 1 fold & AA = 1 fold, during switching OFF conditions. Numbers along the bold lines indicate magnitude of activation or inhibition in terms of the corresponding Hill function values, which lie between 0 and 1. Here AKTp positive feedback on IRS (= 0.50), AKT crosstalk PDE3 (=1) and PDE3 degradation of cAMP (= 0.74) are dominant like in Figure S6. Although increased AKT levels are also increasing S6K1p inhibition of IRS (=0.81) and high FA level is also strengthening PKC inhibition on IRS (=0.48), they are not able to inhibit Insulin signalling pathways due to the dominant AKT-IRS-PI3K positive feedback loop. Contribution of AKT (=0.89) is more than PKA (0.05) to the overall metabolic state, making it highly anabolic.

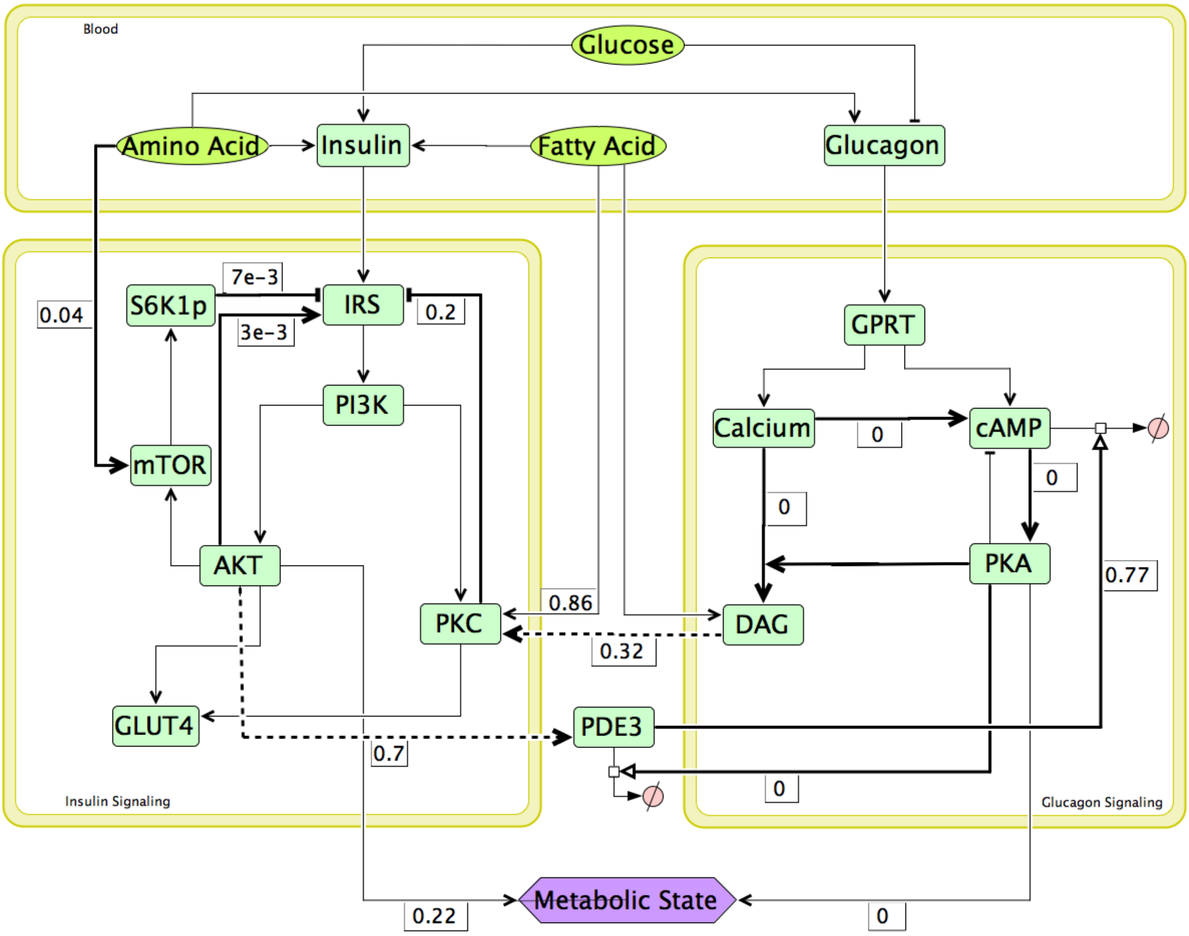

Figure S9. Network map for high FA and high glucose levels in plasma; with glucose = 1.5 folds, AA = 1 fold & FA = 4 folds. Numbers along the bold lines indicate magnitude of activation or inhibition in terms of the corresponding Hill function values, which lie between 0 and 1. Here glucagon signalling module is almost inactive due to inhibition by glucose and highly active cAMP degradation by AKTp via PDE3 (=0.77). Insulin signalling fluxes are only slightly active (in spite of increased insulin secretion by higher glucose and fatty acid levels) due to inhibition by high FA levels via PKC activation (=0.86 vs 0.09 in Figure S7). Moreover inactivated positive feedback of AKT on IRS (=3e-3) is unable to strengthen the anabolic response. Hence, overall metabolic state is only slightly anabolic with contribution from insulin signalling being more than glucagon signalling to the net metabolic state (AKT contribution = 0.22 and PKA contribution = 0).

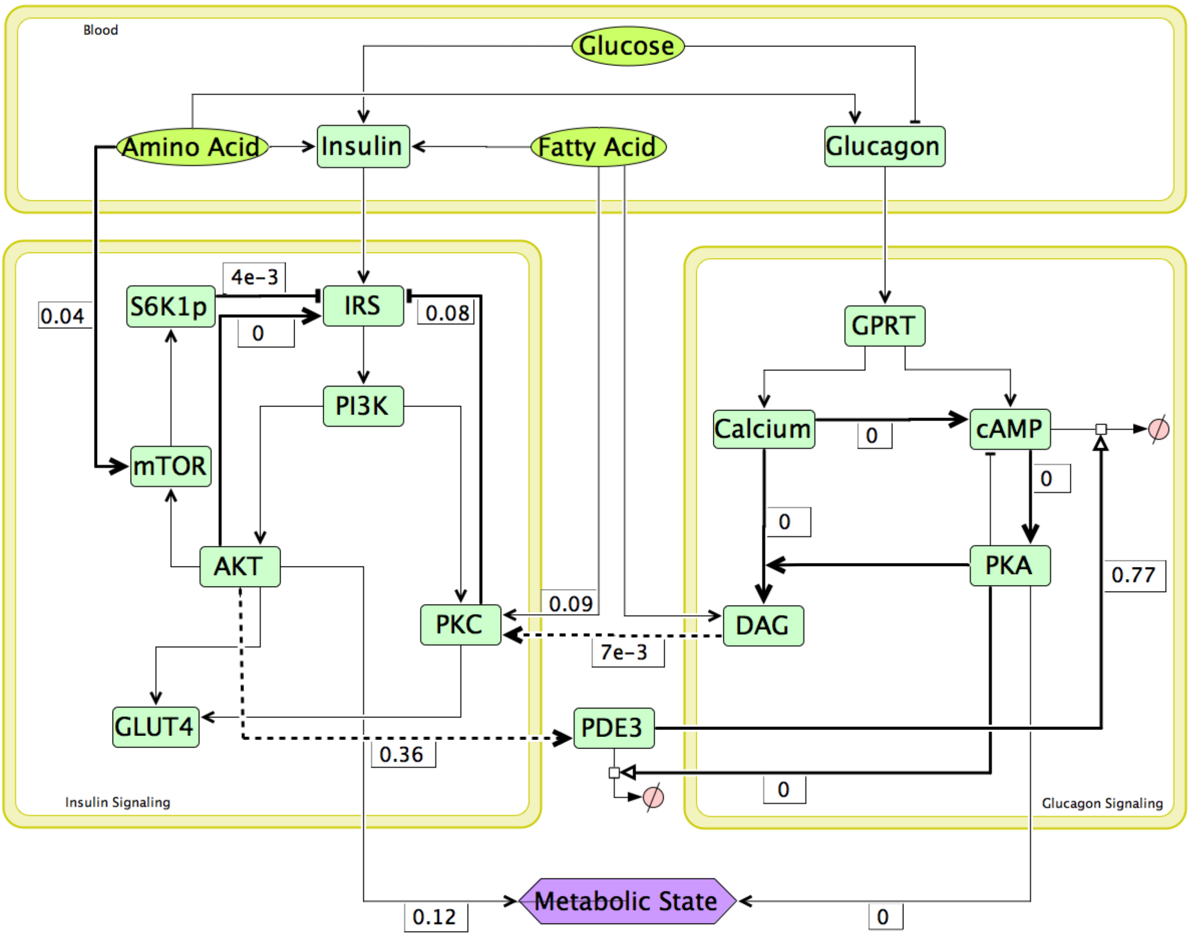

Figure S10. Network map for knockout of AKTp positive feedback on IRS; with glucose = 1.5 folds, AA = 1 fold & FA = 1 fold. Numbers along the bold lines indicate magnitude of activation or inhibition in terms of the corresponding Hill function values, which lie between 0 and 1. Glucagon signalling module is inactive due to inhibition by glucose and PDE3 degradation of cAMP (= 0.77). Insulin signalling module is only slightly active (as AKTp positive feedback on IRS is knocked off). Contribution of AKT (= 0.12) is more than PKA (0) to the overall metabolic state, making it slightly anabolic.

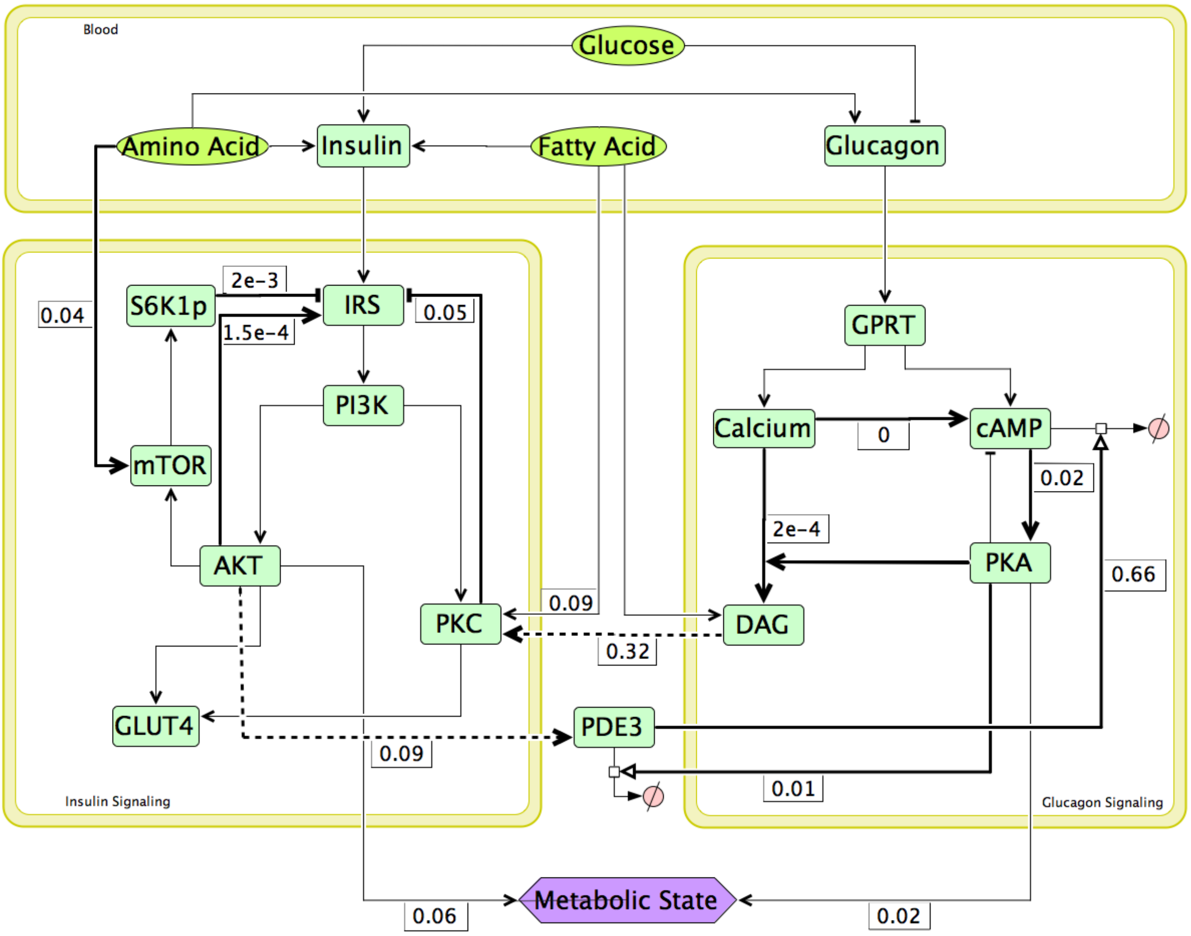

Figure S11. Network map for knockout of Ca positive feedback on cAMP; with glucose = 0.9 folds, AA = 1 fold & FA = 1 fold. Numbers along the bold lines indicate magnitude of activation or inhibition in terms of the corresponding Hill function values, which lie between 0 and 1. Here glucagon signalling module and insulin signalling module are both almost inactive leading to homeostatic conditions even at subnormal glucose levels. Knocking off positive feedback of calcium on cAMP and PDE3 degradation of cAMP (=0.66) are deactivating glucagon signalling fluxes. Contribution of both AKT (= 0.06) and PKA (0.02) are minimal to the overall metabolic state, making the response homeostatic.

Appendix IV : Sensitivity Analysis

We performed sensitivity analysis for 137 model parameters and Initial conditions. The sensitivity indices for 10 most sensitive parameters are reported in table 3 given below.

Table 3 : Sensitivity Index for 10 most sensitive parameters

Rank	Description	Parameter	Sensitivity Index (SI)
1	Vmax for PDE3 degradation of cAMP	kc2	-0.1720
2	Rate constant for AKTp dephosphorelation	k4b1	0.1612
3	Half saturation constant of PKA activation by cAMP	kcamp1	-0.1540
4	Rate constant for PI3Kp inactivation	k4b	0.1325
5	Rate constant of AKT activation	k4f1	-0.1305
6	Half saturation constant for glucose dependent insulin secretion	k_Glu	0.1300
7	Rate constant for IRS dephosphorelation	k2b	0.1256
8	Glucose dependent glucagon secretion constant	Gm	0.1210
9	Rate constant for PI3K activation	k4f	0.1201
10	Half saturation constant for cAMP activation by Glucagon	kcm1	0.1194

Signalling events related to cAMP, IRS, PI3K & AKT are most sensitive in the network. Among feedbacks & cross talks, positive feedbacks of AKT on IRS is most sensitive. Initial conditions of Insulin signalling variable like IR, IRS, PI3K & AKT have slightly significant sensitivity indices as compared to other variables which are equal to zero in their range of robustness. The SI's in these signalling components indicate that variability in the associated rates could produce larger variability in bistable responses. Changing the most sensitive parameter 'kc2' led to doubling of the Ps in the range of robustness.

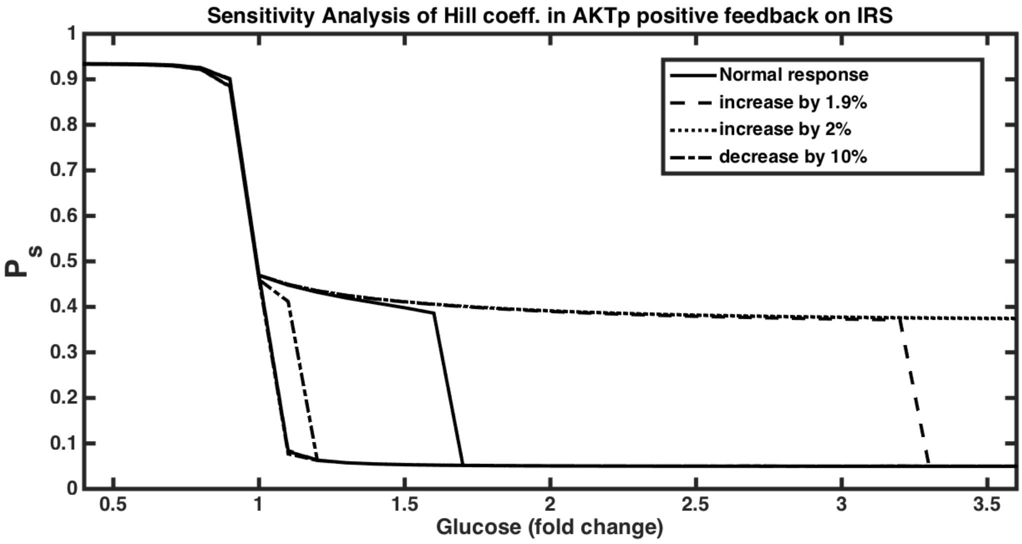

(a)

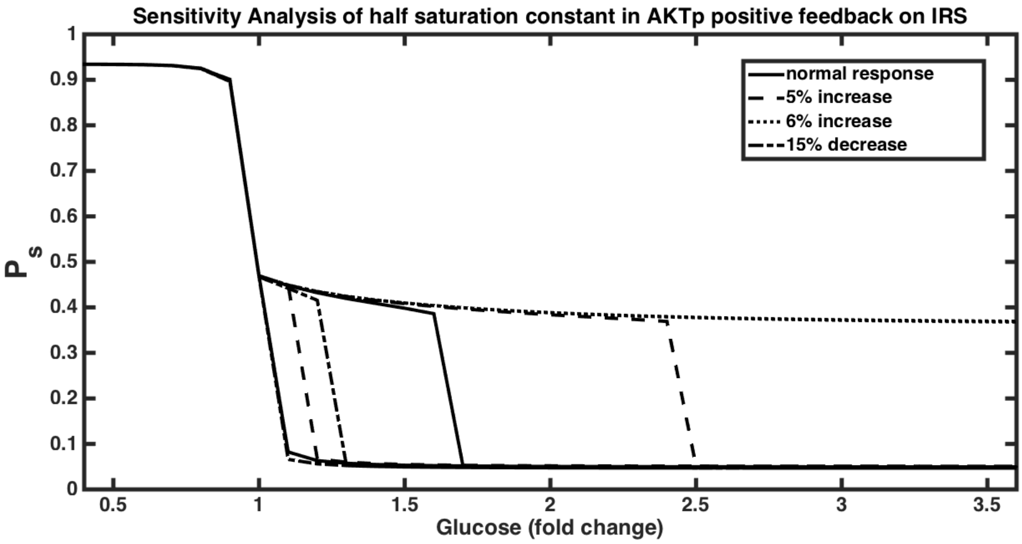

(b)

Figure S12. Sensitivity Analysis of AKTp positive feedback on IRS. (a) As the value of hill coefficient is increased up to 1.9%, bistability span increases in the anabolic space. Beyond 2% increase response turns monostable and homeostatic. While on decreasing, bistability span decreases up to 10% and beyond that it turns monostable. (b) As the value of half saturation constant is increased up to 5%, bistability span increases in the anabolic space. Beyond 6% increase response turns monostable and homeostatic. While on decreasing, bistability span decreases upto 15% and beyond that it turns monostable.

Given the importance of this feedback on the bistable response we have also performed sensitivity analysis of the hill function representing the positive feedback of AKTp on IRS (Supplementary Figure 12 (a) & (b)). For the hill coefficient parameter n, bistable response is maintained for changes ranging from -20% to +1.8% and for the half saturation constant k,

bistable response is maintained for changes ranging from -15% to +10%. As the values of n & k are increased, bistability span increased and response turned monostable beyond increase in n = 1.8% & k = 10%. Whereas, as the values of n and k are reduced, bistability span decreases in the anabolic space for n up to 20% and k up to 15% reduction.

Dynamic Response

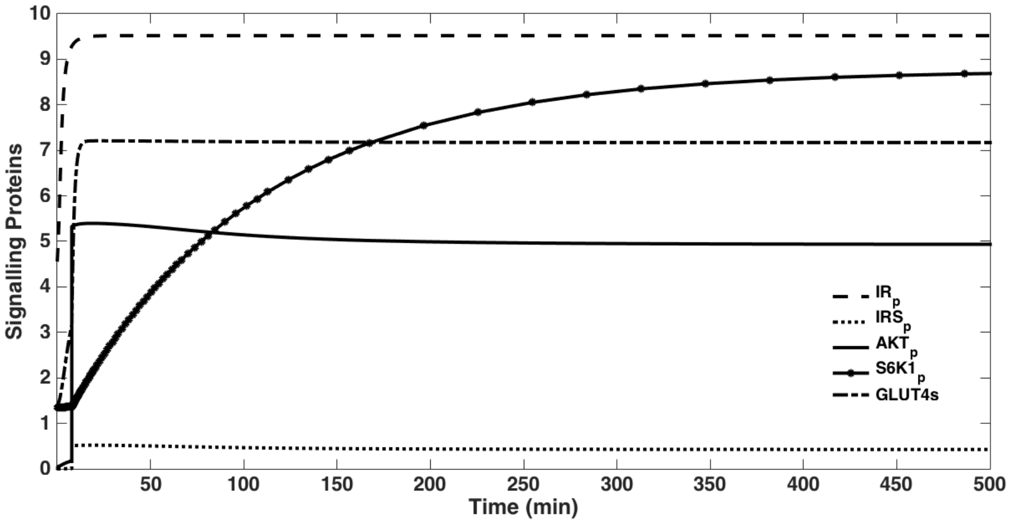

Figure S13. Dynamic response of signaling proteins after changing the macronutrients (Glucose, Fatty Acid and Amino Acid) by a factor of 2. S6K1p and AKTp take the longest time to stabilize (500 and 100 minutes, respectively). Rest of the proteins stabilize within 30 minutes. The steady state simulations were performed for 1000 minutes to ensure that all state variables have reached the steady state.

REFERENCES

1. Lee, J., Pilch, P. F., Shoelson, S. E. & Scarlata, S. F. Conformational changes of the insulin receptor upon insulin binding and activation as monitored by fluorescence spectroscopy. *Biochemistry* (1997). doi:10.1021/bi961815g
2. Baron, V. *et al.* Insulin Binding to Its Receptor Induces a Conformational Change in the Receptor C-Terminus. *Biochemistry* (1990). doi:10.1021/bi00471a019
3. Boura-Halfon, S. & Zick, Y. Phosphorylation of IRS proteins, insulin action, and insulin resistance. *American Journal of Physiology - Endocrinology and Metabolism* (2009). doi:10.1152/ajpendo.90437.2008
4. Hemmings, B. A. & Restuccia, D. F. The PI3k-PKB/Akt pathway. *Cold Spring Harbor Perspectives in Biology* (2015). doi:10.1101/cshperspect.a026609
5. Taniguchi, C. M., Emanuelli, B. & Kahn, C. R. Critical nodes in signalling pathways: insights into insulin action. *Nat. Rev. Mol. Cell Biol.* **7**, 85–96 (2006).
6. Toker, A. Protein kinases as mediators of phosphoinositide 3-kinase signaling. *Mol. Pharmacol.* (2000).
7. Liu, Y. F. *et al.* Insulin stimulates PKCζ-mediated phosphorylation of insulin receptor substrate-1 (IRS-1). A self-attenuated mechanism to negatively regulate the function of IRS proteins. *J. Biol. Chem.* (2001). doi:10.1074/jbc.M007281200
8. Ravichandran, L. V., Esposito, D. L., Chen, J. & Quon, M. J. Protein Kinase C-ζ

Phosphorylates Insulin Receptor Substrate-1 and Impairs Its Ability to Activate Phosphatidylinositol 3-Kinase in Response to Insulin. *J. Biol. Chem.* (2001). doi:10.1074/jbc.M007231200
9. Tanti, J. F. *et al.* Alteration in insulin action: Role of IRS-1 serine phosphorylation in the retroregulation of insulin signalling. in *Annales d'Endocrinologie* (2004). doi:10.1016/S0003-4266(04)95629-6
10. Gual, P., Le Marchand-Brustel, Y. & Tanti, J.-F. Positive and negative regulation of insulin signaling through IRS-1 phosphorylation. *Biochimie* (2005). doi:10.1016/j.biochi.2004.10.019
11. Paz, K. *et al.* Phosphorylation of insulin receptor substrate-1 (IRS-1) by protein kinase B positively regulates IRS-1 function. *J. Biol. Chem.* (1999). doi:10.1074/JBC.274.40.28816
12. Bryant, N. J., Govers, R. & James, D. E. Regulated transport of the glucose transporter GLUT4. *Nature Reviews Molecular Cell Biology* (2002). doi:10.1038/nrm782
13. Chang, L. & Chiang, S.-H. Insulin signaling and the regulation of glucose transport. *Mol. Med.* (2006). doi:10.2119/2005-00029.Saltie
14. Roos, S., Lagerlöf, O., Wennergren, M., Powell, T. L. & Jansson, T. Regulation of amino acid transporters by glucose and growth factors in cultured primary human trophoblast cells is mediated by mTOR signaling. *Am. J. Physiol. - Cell Physiol.* (2009). doi:10.1152/ajpcell.00191.2009
15. Wu, Q. *et al.* FATP1 Is an Insulin-Sensitive Fatty Acid Transporter Involved in Diet-Induced Obesity. *Mol. Cell. Biol.* (2006). doi:10.1128/mcb.26.9.3455-3467.2006
16. Yoon, M.-S. The Role of Mammalian Target of Rapamycin (mTOR) in Insulin Signaling. *Nutrients* (2017). doi:10.3390/nu9111176
17. Giri, L., Mutalik, V. K. & Venkatesh, K. V. A steady state analysis indicates that negative feedback regulation of PTP1B by Akt elicits bistability in insulin-stimulated GLUT4 translocation. *Theor. Biol. Med. Model.* (2004). doi:10.1186/1742-4682-1-2
18. Vinod, P. K. U. & Venkatesh, K. V. Quantification of the effect of amino acids on an integrated mTOR and insulin signaling pathway. *Mol. Biosyst.* (2009). doi:10.1039/b816965a
19. DiPilato, L. M. *et al.* The Role of PDE3B Phosphorylation in the Inhibition of Lipolysis by Insulin. *Mol. Cell. Biol.* (2015). doi:10.1128/mcb.00422-15
20. Mutalik, V. K. & Venkatesh, K. V. Quantification of the glycogen cascade system: The ultrasensitive responses of liver glycogen synthase and muscle phosphorylase are due to distinctive regulatory designs. *Theor. Biol. Med. Model.* (2005). doi:10.1186/1742-4682-2-19
21. Brubaker, P. L. & Drucker, D. J. Structure-function of the glucagon receptor family of G protein-coupled receptors: The glucagon, GIP, GLP-1, and GLP-2 receptors. *Receptors and Channels* (2002). doi:10.1080/10606820213687
22. Gether, U. Uncovering molecular mechanisms involved in activation of G protein-coupled receptors. *Endocr. Rev.* **21**, 90–113 (2000).
23. O'Neill, P. R. *et al.* The structure of dynamic GPCR signaling networks. *Wiley Interdiscip. Rev. Syst. Biol. Med.* (2014). doi:10.1002/wsbm.1249
24. Rosenbaum, D. M., Rasmussen, S. G. F. & Kobilka, B. K. The structure and function of G-protein-coupled receptors. *Nature* (2009). doi:10.1038/nature08144
25. Skalhegg, B. S. & Tasken, K. Specificity in the cAMP/PKA signaling pathway. Differential expression,regulation, and subcellular localization of subunits of PKA. *Frontiers in bioscience : a journal and virtual library* (2000).
26. Berridge, M. J. Inositol trisphosphate and calcium signalling mechanisms. *Biochimica et Biophysica Acta - Molecular Cell Research* (2009).

doi:10.1016/j.bbamcr.2008.10.005
27. Szendroedi, J. *et al.* Role of diacylglycerol activation of PKCθ in lipid-induced muscle insulin resistance in humans. *Proc. Natl. Acad. Sci.* (2014). doi:10.1073/pnas.1409229111
28. Dalla Man, C., Rizza, R. A. & Cobelli, C. Meal simulation model of the glucose-insulin system. *IEEE Trans. Biomed. Eng.* **54**, 1740–1749 (2007).
29. Riccobene, T. A., Omann, G. M. & Linderman, J. J. Modeling activation and desensitization of G-protein coupled receptors provides insight into ligand efficacy. *J. Theor. Biol.* (1999). doi:10.1006/jtbi.1999.0988
30. Nyman, E. *et al.* A hierarchical whole-body modeling approach elucidates the link between in vitro insulin signaling and in vivo glucose homeostasis. *J. Biol. Chem.* (2011). doi:10.1074/jbc.M110.188987
